# The role of self-esteem as moderator of the relationship between experienced stigma and anxiety and depression among tuberculosis patients

**DOI:** 10.1038/s41598-023-34129-4

**Published:** 2023-04-27

**Authors:** Xu Chen, Yunting Chen, Ling Zhou, Jiao Tong

**Affiliations:** 1 Lianyungang Maternal and Child Health Hospital, NO. 669 Qindongmen Street, Haizhou District, Lianyungang, 222000 Jiangsu People’s Republic of China; 2 School of Public Health, Dalian Medical University, 9 Western Section, Lvshun South Street, Lvshunkou District, Dalian, 116044 Liaoning People’s Republic of China

**Keywords:** Psychology, Diseases

## Abstract

Anxiety and depression are very common in tuberculosis (TB) patients and can adversely affect TB treatment adherence, ultimately leading to higher morbidity, mortality and drug resistance. Therefore, the aim of this study was to identify the association among experienced stigma, self-esteem and anxiety and depression, and to further explore whether self-esteem could be a moderator in the association between experienced stigma and anxiety and depression in TB patients. A total of 473 TB patients from Dalian, Liaoning Province, Northeast China participated in a cross-sectional survey. A structured questionnaire was developed to collect data. Hierarchical multiple regression was used to analyze the association among experienced stigma, self-esteem and experienced stigma × self-esteem interaction with anxiety and depression. Simple slope analysis was applied to visualize the interaction. Experienced stigma was positively associated with anxiety (*B* = 0.307, *P* < 0.01) and depression (*B* = 0.277, *P* < 0.01), and self-esteem was negatively associated with anxiety (*B* = − 0.215, *P* < 0.01) and depression (*B* = − 0.351, *P* < 0.01) in TB patients. The association between experienced stigma and anxiety was different in the low (1 standard deviation (SD) below the mean, *B* = 0.376, standard error (SE) = 0.056, *P* < 0.01) and high (1 SD above the mean, *B* = 0.228, SE = 0.060, *P* < 0.01) groups of self-esteem. Additionally, the association between experienced stigma and depression was also different in the low (1 SD below the mean, *B* = 0.363, SE = 0.053, *P* < 0.01) and high (1 SD above the mean, *B* = 0.179, SE = 0.056, *P* < 0.01) groups of self-esteem. Self-esteem could moderate the association between experienced stigma and anxiety and depression. In addition to reducing experienced stigma, enhancing self-esteem as a way to reduce the impact of experienced stigma on anxiety and depression can also help improve the mental health of TB patients.

## Introduction

Tuberculosis (TB), caused by *Mycobacterium tuberculosis* (MTB), is a chronic infectious disease that poses a serious threat to human health^1^. Although TB can be cured through timely diagnosis and treatment, it remains a major global public health problem. The reason is that it places a serious burden on morbidity and mortality worldwide, especially in vulnerable populations^1,2^. An estimated 9.9 million people were infected with TB globally, and 1514,000 people died from TB in 2020^1^. China is one of the three countries with the largest TB burden, accounting for 8.5% of the world’s TB cases in 2020^1^. While significant progress has been made in efforts to end TB, key issues facing TB patients remain. The course and prognosis of TB are influenced not only by clinical characteristics but also by psychological characteristics^3^. Globally, mental health issues remain an important barrier to effective TB treatment and prevention initiatives^4^.

Various studies in different countries have confirmed a high prevalence of anxiety and depression in TB patients. The prevalence of anxiety ranged from 13.5 to 65.0%, and depression from 29.8 to 61.1%^5–9^. This is significantly higher than in the general population and patients with other physical conditions^10,11^. TB patients with anxiety and depression can reduce treatment adherence and also affect patients’ well-being and quality of life^6,12^. However, reduced adherence often leads to poor treatment outcomes, which not only increases mortality and the emergence of drug-resistant TB, but also contributes to sustained community TB transmission and increased public health system spending^13,14^. Thus, anxiety and depression may be the silent drivers of the TB epidemic and the emergence of drug-resistant TB, which poses a major challenge to the global elimination of TB. However, anxiety and depression are treatable with appropriate interventions. In India, psychological interventions during treatment for TB patients significantly improved anti-TB treatment adherence^15^. Given the prevalence, detrimental effects and treatability of anxiety and depression in TB patients, its related factors and potential mechanisms deserve more attention and further exploration to identify populations that need focus and develop effective interventions. Previous studies have reported that several factors affect the level of anxiety and depression in TB patients, such as age^16^, gender^3^, education^11^, annual household income^9^, type of TB^17^, stage of treatment^18^, adverse drug reactions^19^ and comorbidities of chronic disease^20^.

Stigma was defined by Goffman as an attribute linking a person to a set of undesirable traits that may lead to prejudice and discrimination^21^. It was part of the illness experience of many chronic diseases^22^. Among TB patients, it was common to avoid contact with family members, especially children, for fear of spreading the disease^23^. Furthermore, stigma was prevalent in the community, where TB patients were often stigmatized and ostracized by community members^23,24^. In a community-based survey, 73% of people reported stigmatizing TB patients^25^. Studies have also indicated that more than 50% of TB patients often suffer from stigma^26^. Many evidence has shown that stigma can be an important risk factor for depression and anxiety in multiple patient groups, such as people living with human immunodeficiency virus (HIV) and overweight adolescents^27,28^. Perceived stigma was also identified to be significantly associated with anxiety and depression among TB patients in Southwest Ethiopia^18^. However, stigma was found to only predict anxiety and not depression in adults with new-onset epilepsy^29^. Thus, experienced stigma may play an important negative role in the development of anxiety and depression among TB patients in Northeast China.

Self-esteem is an aspect of an individual’s self-concept, which reflects an individual’s overall evaluation and feeling of their own value at any point in time^29^. Self-esteem affects a person’s ability to adapt to new or different situations and is an important resource for individuals to cope with stress and maintain their health^30^. Vulnerability models argues that low self-esteem is a risk factor for the development of depression^31^. Terror management theory states that self-esteem can act as a buffer to relieve one’s anxiety^32^. Previous research has also confirmed that high self-esteem is associated with fewer psychological problems. For example, studies in children of HIV-infected parents have shown that self-esteem is a predictor of anxiety and depression^33^. Studies in multidrug resistant tuberculosis (MDR-TB) patients have elucidated a significant association between self-esteem and anxiety and depression^34^. Of note here, the emotion-regulation model of self-esteem describes that self-esteem can moderate the associations between negative events and emotional reactions^35^. Specifically, when individuals face significant stressors, high self-esteem will protect individuals from psychological distress, while low self-esteem will put individuals at greater risk of psychological distress. Empirical studies further demonstrate that self-esteem can buffer the harmful association between stigma and psychological distress in infertile men^36^. Based on these, we deduced that self-esteem not only had a direct effect on anxiety and depression, but also might be a positive resource in buffering the association between experienced stigma and anxiety and depression in TB patients.

Over the years, there have been numerous studies on the effects of stigma on anxiety and depression, but few studies have explored whether self-esteem moderates the association between stigma and anxiety and depression^22^. Moreover, to our knowledge, no study has simultaneously explored the association between experienced stigma, self-esteem and anxiety and depression among TB patients in Northeast China. Therefore, based on the above conceptual framework and literature, we conducted a cross-sectional survey in Dalian, Liaoning Province, Northeast China to verify the following six hypotheses among TB patients:

### Hypothesis 1

Experienced stigma is positively related to anxiety.

### Hypothesis 2

Experienced stigma is positively related to depression.

### Hypothesis 3

Self-esteem is negatively related to anxiety.

### Hypothesis 4

Self-esteem is negatively related to depression.

### Hypothesis 5

The association between experienced stigma and anxiety could be moderated by self-esteem.

### Hypothesis 6

The association between experienced stigma and depression could be moderated by self-esteem.

## Methods

### Study design and setting

A cross-sectional survey was carried out between November 2020 and March 2021 in Dalian, Liaoning Province, Northeast China. Three medical institutions serving TB patients were chosen based on the number of patients attending, type of patients and location. The first was the main medical institutions for TB patients in Dalian. It was also the only tertiary specialized hospital for TB prevention and control in Dalian, divided into north and south parts. The south branch, located in Ganjingzi district, serves more critically ill and urban TB patients. The north branch, located in Pulandian district, serves both rural and urban TB patients. The other two medical institutions were located in Lushunkou district and Zhuanghe city (a county-level city), respectively. They were TB dispensaries, which served only local TB patients with a milder instance of the disease.

### Participants

This study recruited TB patients who attended three medical institutions between November 2020 and March 2021 and met the inclusion criteria. Patients with a clear TB diagnosis, aged 18 years or older, and consenting to participate in the study were eligible. Patients who had completed treatment, had psychosis, had communication problems, had difficulty understanding the questionnaire, and could not follow the study procedures were excluded from the study. TB was diagnosed according to the national TB program guidelines. Finally, 481 TB patients were recruited and completed a structured questionnaire. Among them, 8 questionnaires were removed because of logic errors or large amounts of missing data. Thus, the data for 473 TB patients were included in the analysis, with a participation rate of 98.3%.

### Ethics procedure

All methods in our study were conducted in accordance with the Declaration of Helsinki. This study protocol was in accordance with the ethical standards, and was reviewed and approved by the Ethics Committee of Dalian Medical University. Prior to participating in the study, patients were informed of the purpose of the study and other information, and were assured that all information they provided would be confidential. Written informed consent was obtained from each patient in the study.

### Measurement

#### Anxiety and depression

Anxiety and depression were measured using the Kessler Psychological Distress Scale (K-10) (Chinese version)^37^. Previous studies in a variety of populations in China, including TB patients, have demonstrated the reliability and validity of the Chinese version of the K-10^38,39^. The scale was composed of 10 items. It was a self-report tool used to measure nonspecific symptoms of depression and anxiety^40^. The two-factor anxiety and depression structure of the scale has been considered optimal for clinical samples^41^. The anxiety dimension included 4 items, which mainly referred to nervousness and agitation. The depression dimension included 6 items, which mainly referred to fatigue and negative affect (including hopeless, depressed, sad and worthless). Each item was scored using a 5-point Likert scale ranging from 1 (none of the time) to 5 (all the time). The highest score for anxiety was 20, and the highest score for depression was 30. Higher scores reflected higher levels of nonspecific depression and anxiety. The validity of anxiety (comparative fit index (CFI) = 0.954, goodness-of-fit index (GFI) = 0.959, Tucker-Lewis index (TLI) = 0.863, and standardized root mean square residual (SRMR) = 0.049) and depression (CFI = 0.984, GFI = 0.978, TLI = 0.974, and SRMR = 0.022) was confirmed by exploratory and confirmatory factor analyses. Cronbach’s α coefficient of K-10 in this study was 0.842 (anxiety) and 0.896 (depression), respectively.

#### Experienced stigma

Experienced stigma was assessed using a 9-item stigma questionnaire^39^. The questionnaire was developed according to the social and cultural background of China and had good reliability and validity^39^. Each item was rated on a 4-point Likert scale ranging from 1 (strongly disagree) to 4 (strongly agree). The scores for each item were added to give a total score ranging from 9 to 36. A higher score indicated a higher level of experienced stigma. In this study, Cronbach’s α was 0.946. The confirmatory factor analyses for it were CFI = 0.949, GFI = 0.908, TLI = 0.927, and SRMR = 0.034.

#### Self-esteem

The Rosenberg Self-Esteem Scale (RSES) (Chinese version) was used to assess the level of self-esteem of TB patients^42^. Its purpose was to assess an individual’s overall feelings of self-worth and self-acceptance. The Chinese version of the RSES has been widely used and has been shown to have a stable factor structure across diverse populations^38^. The scale was consisted of 10 items. Each item was scored on a 4-point Likert scale ranging from 1 (strongly disagree) to 4 (strongly agree). The total score ranged from 10 to 40. The higher the score, the higher the self-esteem is. In our study, it has a high internal consistency (Cronbach’s α = 0.880). The confirmatory factor analyses for RSES were CFI = 0.956, GFI = 0.946, TLI = 0.920, and SRMR = 0.069.

#### Demographic characteristics

Gender, age and residence were collected in this study. Age was divided into three ranges including 18–30, 31–44, and ≥ 45. Options for residence included “urban” and “rural”.

#### Treatment status variables

Three treatment status factors were assessed, including treatment category, comorbid illness and adverse drug reactions. Treatment category was divided into “new” and “relapse”. Comorbid illness was assessed by asking “Do you currently have any other medical conditions?”. Adverse drug reactions were obtained by asking “Have you had any adverse reactions during taking the drug?”.

### Statistical analysis

The collected questionnaires were checked for completeness and consistency. Qualified questionnaires were coded and entered into EpiData 3.1 (EpiData Association, Odense, Denmark) software and exported to SPSS 21.0 (IBM Corporation, Armonk, State of New York) for statistical analysis. Descriptive statistical analysis was performed to describe demographic characteristics and treatment status. Skewness and kurtosis were utilized to test the normality of the variables. The absolute values of skewness and kurtosis of the studied variables were all within 0.6 and 1.1, respectively, indicating that normal distribution was satisfied^43^. Mean and standard deviation (SD) were calculated for continuous variables, and frequency and percentage were used for categorical variables. The *t*-test or one-way ANOVA was used to compare the differences in anxiety and depression among patients with different demographic characteristics and treatment status. Pearson correlation analysis was used to evaluate the correlation among continuous variables. In this study, variance inflation factor (VIF) values < 10 and tolerance > 0.1 indicated that multicollinearity was not a problem in the estimate^44^. Hierarchical multiple regression analysis was applied to examine the association among experienced stigma, self-esteem and anxiety and depression as well as to explore the moderating role of self-esteem on the association among experienced stigma and anxiety and depression. In step 1, gender, age and potential controlling variables related to anxiety and depression in the univariate analysis were added. Experienced stigma and self-esteem were added in step 2. Obviously, the product of experienced stigma and self-esteem was added in step 3. All study variables were standardized before regression analysis to reduce the potential effects of multicollinearity^45^. If the interaction effect was statistically significant, Aiken and West’s procedures were followed with a simple slope analysis to visualize the interaction term^46^. All of the above statistical analysis were two-sided tests, and *P* < 0.05 was considered statistically significant.

## Results

### Demographic characteristics and treatment status

A total of 473 TB patients were included in this study, with a mean age of 48.36 ± 17.58 years, and most patients (60.0%) were 45 years or above. Male (69.1%) and rural (66.8%) patients had a higher proportion. A small number of patients (15.2%) were relapse, and more than one-third (34.5%) had other medical conditions. In the course of taking medication, 31.9% of patients had adverse drug reactions. In this study, the mean scores of anxiety dimension and depression dimension were 8.02 ± 3.17 and 11.60 ± 4.65, respectively. Univariate analysis indicated that there were significant differences in anxiety and depression among patients with different ages, treatment categories, comorbidities and adverse drug reactions (*P* < 0.05) (Table 1).

**Table 1:** Demographic characteristics and treatment status of TB patients and univariate analysis for the factors in relation to anxiety and depression.

Variables	n (%)	Anxiety	*df*	*t*/*F*-value	*P*-value	Effect size	Depression	*df*	*t*/*F*-value	*P*-value	Effect size
		Mean ± SD					Mean ± SD				
Gender											
Male	327 (69.1)	7.92 ± 3.22	471	− 1.003	0.317	0.100	11.63 ± 4.71	471	0.200	0.842	0.020
Female	146 (30.9)	8.24 ± 3.04					11.53 ± 4.54				
Age (years)											
18–30	107 (22.6)	7.28 ± 2.83	472	4.270	**0.015**	0.018	10.30 ± 3.78	472	5.689	**0.004**	0.024
31–44	82 (17.3)	7.95 ± 3.24					11.71 ± 4.91				
≥ 45	284 (60.0)	8.32 ± 3.23					12.06 ± 4.79				
Residence											
Rural	316 (66.8)	8.09 ± 3.19	471	0.687	0.492	0.067	11.87 ± 4.58	471	1.830	0.068	0.179
Urban	157 (33.2)	7.88 ± 3.13					11.04 ± 4.75				
Treatment category											
New	401 (84.8)	7.88 ± 3.10	471	2.374	**0.018**	0.305	11.39 ± 4.53	471	2.349	**0.019**	0.301
Relapse	72 (15.2)	8.83 ± 3.42					12.78 ± 5.15				
Comorbid illness											
Yes	163 (34.5)	8.92 ± 3.17	471	4.569	** < 0.001**	0.449	13.00 ± 4.70	471	4.867	** < 0.001**	0.472
No	310 (65.5)	7.55 ± 3.07					10.86 ± 4.46				
Adverse drug reactions											
Yes	151 (31.9)	8.83 ± 3.08	471	3.880	** < 0.001**	0.383	12.77 ± 4.54	471	3.823	** < 0.001**	0.378
No	322 (68.1)	7.64 ± 3.14					11.05 ± 4.60				

Significant values are in bold.

*SD* standard deviations, *df* degrees of freedom.

### Correlations among continuous variables

The correlations among age, experienced stigma, self-esteem, anxiety and depression are displayed in Table 2. Experienced stigma was positively correlated with anxiety and depression (*r* = 0.413 and *r* = 0.449, respectively, *P* < 0.01). This supported hypotheses 1 and 2. Self-esteem was negatively correlated with anxiety and depression (*r* = − 0.371 and *r* = − 0.494, respectively, *P* < 0.01). This supported hypotheses 3 and 4. Additionally, age was positively correlated with depression and anxiety (*r* = 0.113, *P* < 0.05 and *r* = 0.140, *P* < 0.01, respectively), and experienced stigma was negatively correlated with self-esteem (*r* = − 0.441, *P* < 0.01).

**Table 2:** Correlations among age, experienced stigma, self-esteem, anxiety and depression.

Variables	Mean ± SD	Correlations (*r*)				
		1	2	3	4	5
1. Age (years)	48.36 ± 17.58	1				
2. Experienced stigma	18.86 ± 7.14	0.206**	1			
3. Self-esteem	30.15 ± 4.49	− 0.145**	− 0.441**	1		
4. Anxiety	8.02 ± 3.17	0.113*	0.413**	− 0.371**	1	
5. Depression	11.60 ± 4.65	0.140**	0.449**	− 0.494**	0.832**	1

*SD* standard deviations.

*P < 0.05.

**P < 0.01 (two-tailed).

### Hierarchical regression analysis

The results of hierarchical regression analysis are presented in Tables 3 and 4. First, the linear combination of demographic characteristics and treatment status controlling variables (gender, age, treatment category, comorbid illness and adverse drug reactions) significantly explained anxiety (*F* = 8.174, *R*^2^ = 0.080, *P* < 0.001) and depression (*F* = 8.339, *R*^2^ = 0.082, *P* < 0.001).

**Table 3:** Results of hierarchical multiple regression analysis on anxiety.

Variables	Step 1 (*B*)	Step 2 (*B*)	Step 3 (*B*)
Gender	0.115	0.221*	0.222*
Age (years)	0.007	− 0.056	− 0.053
Treatment category	− 0.318*	− 0.191	− 0.190
Comorbid illness	− 0.368***	− 0.307**	− 0.302**
Adverse drug reactions	− 0.351***	− 0.258**	− 0.241**
Experienced stigma		0.307***	0.302***
Self-esteem		− 0.215***	− 0.220***
Experienced stigma × Self-esteem			− 0.074*
*F*	8.174***	23.794***	21.478***
*df*	472	472	472
*R*^2^	0.080	0.264	0.270
Adjusted* R*^2^	0.071	0.253	0.258
Δ*R*^2^	0.080	0.183	0.007

*df* degrees of freedom.

*P < 0.05.

**P < 0.01.

***P < 0.001 (two-tailed).

**Table 4:** Results of hierarchical multiple regression analysis on depression.

Variables	Step 1 (*B*)	Step 2 (*B*)	Step 3 (*B*)
Gender	0.000	0.120	0.121
Age (years)	0.025	− 0.044	− 0.040
Treatment category	− 0.299*	− 0.118	− 0.117
Comorbid illness	− 0.384***	− 0.307***	− 0.301***
Adverse drug reactions	− 0.332**	− 0.225**	− 0.204*
Experienced stigma		0.277***	0.271***
Self-esteem		− 0.351***	− 0.357***
Experienced stigma × Self-esteem			− 0.092**
*F*	8.339***	35.163***	32.091***
*df*	472	472	472
*R*^2^	0.082	0.346	0.356
Adjusted* R*^2^	0.072	0.336	0.345
Δ*R*^2^	0.082	0.264	0.010

*df* degrees of freedom.

*P < 0.05.

**P < 0.01.

***P < 0.001 (two-tailed).

**Figure 1:**
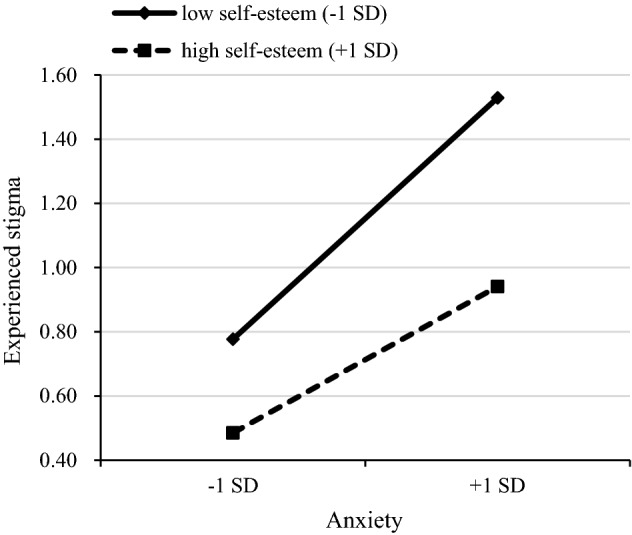
Simple slope plot of interaction between experienced stigma and self-esteem on anxiety. *SD* standard deviations.

**Figure 2:**
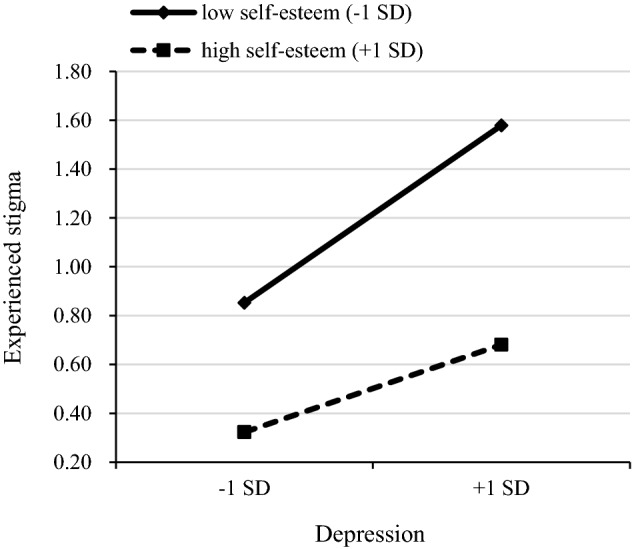
Simple slope plot of interaction between experienced stigma and self-esteem on depression. *SD* standard deviations.

In the second step, adding experienced stigma and self-esteem improved the model fit of anxiety (*F* = 23.794, *R*^2^ = 0.264, Δ*R*^2^ = 0.183, *P* < 0.001) and depression (*F* = 35.163, *R*^2^ = 0.346, Δ*R*^2^ = 0.264, *P* < 0.001). Experienced stigma exhibited significant main effect on anxiety (*B* = 0.307, *P* < 0.001) and depression (*B* = 0.277, *P* < 0.001), and self-esteem also exhibited a significant main effect on anxiety (*B* = − 0.215, *P* < 0.001) and depression (*B* = -0.351, *P* < 0.001). This supported hypotheses 1–4. For the anxiety model, the experienced stigma × self-esteem interaction term significantly explained an additional 0.7% of the variance (*F* = 21.478, *R*^2^ = 0.270, Δ*R*^2^ = 0.007, *P* < 0.001) in the third step. The interaction term was negatively correlated with anxiety (*B* = − 0.074, *P* < 0.05), suggesting that self-esteem plays a moderating role between experienced stigma and anxiety. We followed Aiken and West’s procedures and plotted the relationship under low (1 SD below the mean) and high (1 SD above the mean) levels of self-esteem. Simple slope analysis showed that the impacts of experienced stigma on anxiety were different between the low (*B* = 0.376, standard error (SE) = 0.056, *P* < 0.001) and high (*B* = 0.228, SE = 0.060, *P* < 0.001) groups of self-esteem. In other words, when the self-esteem is lower, the relationship between experienced stigma and anxiety becomes stronger (Fig. 1). This supported hypothesis 5. For the depression model, the interaction term also significantly explained an additional 1.0% of the variance (*F* = 32.091, *R*^2^ = 0.356, Δ*R*^2^ = 0.010, *P* < 0.001) in the third step. The interaction term was negatively correlated with depression (*B* = − 0.092, *P* < 0.01), suggesting that self-esteem plays a moderating role between experienced stigma and depression. Simple slope analysis revealed that the impacts of experienced stigma on depression were different between the low (*B* = 0.363, SE = 0.053, *P* < 0.001) and high (*B* = 0.179, SE = 0.056, *P* < 0.01) groups of self-esteem. This supported hypothesis 6 (Fig. 2).

## Discussion

Psychological distress expressed in terms of anxiety and depression is common in TB patients^47^. This study explored the association among experienced stigma, self-esteem and anxiety and depression, as well as examined whether self-esteem could moderate the relationship between experienced stigma and anxiety and depression among TB patients in Dalian, Liaoning Province, Northeast China. To our knowledge, this is the first study in Northeast China to investigate the potential mechanisms underlying the association between experienced stigma and anxiety and depression in TB patients. In addition, the research could help improve psychological adjustment interventions. Our results supported all research hypotheses, revealing that experienced stigma was positively correlated with anxiety and depression, and self-esteem was negatively correlated with anxiety and depression in TB patients. Self-esteem alleviated the relationship between experienced stigma and anxiety and depression. This study is unique in that our findings extend previous studies on the independent effects of experienced stigma on anxiety and depression, highlighting the role of self-esteem in the association between experienced stigma and anxiety and depression in TB patients.

In this study, we found that female was significantly associated with anxiety. This is consistent with other studies showing that female with TB is more likely to experience anxiety than male^18^. Moreover, a review also revealed that female TB patients had poorer mental health overall^16^. Biological factors like hormonal fluctuations associated with menstruation and pregnancy and social factors like family responsibilities may contribute to female’s anxiety^6,48,49^. Our results also suggested that comorbid illness was a risk factor for depression and anxiety. This is consistent with previous studies conducted in Myanmar^50^. Depression is known to have a high prevalence in chronic diseases, which may contribute to a higher risk of depression in TB patients who also have other medical conditions^51,52^. In addition, the combination treatment of TB and other diseases is more complex, expensive and challenging, which may also adversely affect the mental health of patients to some extent^53^. Previous studies have indicated that patients who experience more side effects from TB treatment are at greater risk of anxiety and depression^50,54^. Our study also found that adverse drug reactions were a predictor of depression and anxiety. Among the participants, 31.9% experienced adverse drug reactions such as nausea, anorexia, rash and joint pain. This can impair their adherence to treatment, which in turn affects their mental health^55,56^. As a result, the focus of psychological interventions for TB patients should be on female and patients with comorbidities. Additionally, timely detection and management of patients suffering from adverse drug reactions is also necessary.

Consistent with previous studies, we found that experienced stigma was an influential factor for anxiety and depression in TB patients^49^. Patients with TB are less accepted by society and the negative reactions of others can leave them feeling socially ignored, excluded and isolated^18^. This often leads to social withdrawal and hiding, which ultimately affects the mental health of the patient^57,58^. Moreover, the problem of ‘face’ concern is particularly prominent in Chinese society^59^. Patients with TB are less likely to disclose their condition and seek help to avoid alienation, loss of social status and face^23,60^. This concealment of the condition and the fear of losing friends, status and face may also exacerbate their psychological distress^61,62^. A large number of studies have been conducted on stigma in TB patients. For instance, a previous study showed that good family functioning and doctor-patient communication had a positive effect on reducing stigma among TB patients^63^. Another study suggested that social support was a protective factor against stigma^26^. In Ethiopia, clubs made up of health workers and TB patients have been successful in reducing stigma and improving treatment outcomes by providing social support to patients^64^. In addition, Goffman theorized that individuals with stigmatized characteristics can resist stigma by manipulating they share with others^21^. Therefore, intervention strategies for anxiety and depression in TB patients should include the elimination of stigma, such as strengthening doctor-patient communication and providing social support to patients.

Positive self-esteem is associated with mental health, and boosting self-esteem may be an important part of TB treatment^65^. Our study also confirmed that self-esteem can play a protective role in the prevention of anxiety and depression in TB patients. We found a significant correlation between self-esteem and both anxiety and depression. This is consistent with previous studies conducted among underprivileged children and adolescents^66,67^. More importantly, our study demonstrated that self-esteem moderated the association between experienced stigma and anxiety and depression in TB patients. In general, self-esteem weakened the negative impact of experienced stigma on anxiety and depression. Specifically, the association between experienced stigma and anxiety and depression was exacerbated at low self-esteem levels, whereas the association was buffered at high self-esteem levels. This result is similar to a previous study in TB patients mostly (92.2%) from rural areas in Eastern China^38^. The possible reason for this result is that patients with low self-esteem have more negative self-awareness, which reduces their ability to effectively deal with painful situations. They often feel insurmountable when they encounter difficulties, leading to poor mental health^68^. However, high self-esteem can provide TB patients with a relatively stable internal resource to cope with stress and buffer the negative impact of stigma on mental health^36^. Moreover, people with high self-esteem help build positive social support networks because they believe in their own social worth. On the contrary, low self-esteem not only adversely affects the access to social support, but also ignores the social support provided by others^69^. Our results clearly indicated that improving self-esteem in TB patients was critical to alleviating the negative effects of stigma on anxiety and depression. Consequently, psychological interventions should include specific strategies to increase the patient’s self-esteem, and TB patients with low self-esteem may be more in need of intervention.

There are some limitations that require further explanation in this study. It is first important to point out that the data in this study are cross-sectional, which limits the possibility of causal inference among the study variables. Longitudinal studies are needed to determine causal relationships between these variables in the future. Second, the participants in this study are all from Dalian, Liaoning Province, Northeast China, which can only represent regions with the same situation. The results should be carefully generalized to regions with different sociocultural and background conditions until the current results are replicated. Third, experienced stigma, self-esteem, anxiety and depression in this study were all measured using self-reported questionnaires rather than diagnostic tools, and patients may have given socially satisfactory responses. In addition, anxiety and depression were measured by asking patients about the frequency of symptoms in the past 30 days. This may have affected our results by recall bias and led to an underestimation of our results. Fourth, the data were collected during the Corona Virus Disease 2019 (COVID-19) pandemic, which may have influenced the results and made them appropriate for the context of public health emergencies. Finally, the study only recruited TB patients who came to health facilities for services, ignoring those who did not receive any treatment. This may make our results somewhat underestimated.

## Conclusion

In summary, experienced stigma was positively associated with anxiety and depression, and self-esteem was negatively associated with anxiety and depression in TB patients. Self-esteem can buffer the negative effects of experienced stigma on anxiety and depression. These findings highlight the important practical implications of strengthening self-esteem in TB patients. Therefore, in addition to reducing experienced stigma, enhancing self-esteem as a way to reduce the impact of experienced stigma on anxiety and depression can also help improve the mental health of TB patients and ultimately reduce the adverse consequences of anxiety and depression.

## Data Availability

The raw data supporting the conclusions of this article will be made available by the corresponding author, without undue reservation.
